# Supplementary material for: Relationship among airborne pollen, sensitization, and pollen food allergy syndrome in Asian allergic children
Source: PeerJ. 2022 Nov 1;10:e14243. doi: 10.7717/peerj.14243 (PMC9635357; doi:10.7717/peerj.14243)
Supplement: Table S1 [file peerj-10-14243-s001.pdf]

STUDY NUMBER_____________**INITIALS**__________ **DATE**__________

Q1 Do you have hay fever? Yes No

If yes do you get symptoms mainly in the:

- ❒ Spring time (February/March to May/June)
- ❒ Summertime (May/June to August)
- ❒ Autumn (October to December)
- ❒ Winter (November to February)
- ❒ All year round

Q2 Have you ever had any allergic reactions to food? Yes No

If no – stop here and hand the questionnaire back to the researcher

Q3 Do you experience reactions to foods all year round Yes No

If no – do you only get reactions to food during your main hay fever season? Yes No

Q4 Which foods do you have a reaction to:

Column A

- ❒ Fruit or vegetables
- ❒ Bean sprouts, salad leaves or herbs
- ❒ Nuts including all tree nuts and peanuts
- ❒ Peeling potatoes or other root vegetables
- ❒ Beans, lentils, chickpeas or other legumes

Column B

- ❒ Milk, eggs, chicken
- ❒ Fish, shellfish
- ❒ Wheat or other cereal
- ❒ Other food

Q5 If reactions are to any food in column A, is your reaction to:

- ❒ Raw foods only
- ❒ Both raw and cooked foods
- ❒ Cooked foods only
- ❒ Not sure

Q6 How quickly do the symptoms occur (tick one)

- ❒ On touching lips
- ❒ On biting or chewing
- ❒ On or up to 5 minutes after swallowing
- ❒ Within 15 minutes of eating
- ❒ Within 30 minutes of eating
- ❒ 1-2 hours after eating
- ❒ 2-4 hours after eating
- ❒ More than 6 hours after eating

Q7 Which of the following symptoms do you have after eating foods to which you are allergic (tick all that apply and grade each symptom from 0-3 (0 = none, 1 = mild, 2 = moderate, 3 = severe):

- ❒ Tingling/numbness of lips/mouth or strange mouth sensation (_____)
- ❒ Intense itching of the lips, mouth, palate or ears (_____)
- ❒ Scratchy/sore throat (_____)
- ❒ Urticarial rash (nettle rash) either on contact (e.g. peeling potatoes) or on eating (_____)
- ❒ Swelling of lips, tongue, mouth or throat (_____)
- ❒ Anaphylaxis (rapid onset of flushing, with severe difficulty in breathing or collapse) (_____)
- ❒ Asthma (_____)
- ❒ Rhinitis (hay fever) (_____)
- ❒ Eczema (_____)
- ❒ Vomiting, Diarrhoea or bloating(_____)
- ❒ Other (_____)
