# Supplementary material for: Relationship among airborne pollen, sensitization, and pollen food allergy syndrome in Asian allergic children
Source: PeerJ. 2022 Nov 1;10:e14243. doi: 10.7717/peerj.14243 (PMC9635357; doi:10.7717/peerj.14243)
Supplement: Table S2 [file peerj-10-14243-s002.docx]

Supplementary Table 2. Summary of definitions

| Allergic disease | Definition |
| --- | --- |
| Pollen food allergy syndrome | Positive response to ‘Does your child or do you feel tingling, numbness, itchiness or swelling in the mouth or throat immediately or within 30 min after eating fresh fruit, vegetables, peanuts, tree nuts, root vegetables and beans’? If yes, specify the causative food. |
| Food allergy | Positive response to ‘Has your child or have you ever had food allergy’? If yes, select one or more from the following allergens: egg, milk, wheat, shrimp, crab, fish or shellfish. |
| Asthma | Positive response to ‘Has your child or have you ever experienced wheezing or whistling in the chest’? |
| Seasonal allergic rhinitis | Positive response to ‘Has your child or have you had **s**easonal allergic rhinitis’? |
| Atopic dermatitis | Positive response to ‘Has your child or have you ever had an itchy rash which comes and goes within the last 6 months’? |
