# Supplementary material for: Relationship among airborne pollen, sensitization, and pollen food allergy syndrome in Asian allergic children
Source: PeerJ. 2022 Nov 1;10:e14243. doi: 10.7717/peerj.14243 (PMC9635357; doi:10.7717/peerj.14243)
Supplement: Supplemental Information 2 [file peerj-10-14243-s004.docx]

Category data

Location (1;Tochigi 2;Fukuoka 3;Busan)

Sex (1;Male, 2; Female)

BA, AR, AC, AD, FA, Ana, PFAS, Pollinosis (1 ;yes,2; no)

Dp, Japanese Cedar, Cypress, Ragweed, Orchard Grass, Birch Alder, Juniper, Pine, Tomato, Peach, Japanese Hop（1;positive,2;negative）
